# Efficacy and Use of Cloth Masks: A Scoping Review

**DOI:** 10.7759/cureus.10423

**Published:** 2020-09-13

**Authors:** Mehr Jain, Sonya T Kim, Chenchen Xu, Heidi Li, Greg Rose

**Affiliations:** 1 Medicine, Faculty of Medicine, University of Ottawa, Ottawa, CAN; 2 Internal Medicine, Faculty of Medicine, University of Ottawa, Ottawa, CAN; 3 Infectious Disease, Queensway Carleton Hospital, Ottawa, CAN

**Keywords:** cloth, masks, personal protective equipment, ppe, covid-19, pandemic, coronavirus, public health

## Abstract

During the coronavirus disease 2019 (COVID-19) pandemic, there has been a global shortage of personal protective equipment (PPE). In this setting, cloth masks may play an important role in limiting disease transmission; however, current literature on the use of cloth masks remains inconclusive. This review aims to integrate current studies and guidelines to determine the efficacy and use of cloth masks in healthcare settings and/or the community. Evidence-based suggestions on the most effective use of cloth masks during a pandemic are presented.

Embase, MEDLINE, and Google Scholar were searched on March 31, 2020, and updated on April 6, 2020. Studies reporting on the efficacy, usability, and accessibility of cloth masks were included. Additionally, a search of guidelines and recommendations on cloth mask usage was conducted through published material by international and national public health agencies.

Nine articles were included in this review after full-text screening. The clinical efficacy of a face mask is determined by the filtration efficacy of the material, fit of the mask, and compliance to wearing the mask. Household fabrics such as cotton T-shirts and towels have some filtration efficacy and therefore potential for droplet retention and protection against virus-containing particles. However, the percentage of penetration in cloth masks is higher than surgical masks or N95 respirators.

Cloth masks have limited inward protection in healthcare settings where viral exposure is high but may be beneficial for outward protection in low-risk settings and use by the general public where no other alternatives to medical masks are available.

## Introduction and background

Disposable surgical face masks (also termed procedure masks) and respirators are essential components of personal protective equipment (PPE) for preventing the transmission of infectious diseases. Both the Canadian and international guidelines highlight the importance of proper usage of PPE among frontline healthcare workers (HCWs) during the current coronavirus disease 2019 (COVID-19) pandemic [1-4]. The shortage of PPE observed worldwide as a result of this pandemic places both HCWs and patients at risk [5,6]. Although guidelines from the World Health Organization (WHO) and Centre for Disease Control and Prevention (CDC) suggest various strategies to optimize the supply of PPE in healthcare settings [4,7], there are limited data on alternatives to surgical masks. In these situations, 3D-printed respirators or community-sourced homemade cloth masks may be potential sources to meet demand in healthcare and community settings. Cloth masks are defined as masks made of cloth or any other fabric that has been previously used to make masks, such as cotton, gauze, silk, or muslin [8]. Surgical masks are certified/rated medical PPE that are fluid-resistant and are effective to protect the wearer from large particles of respiratory secretions known as droplets. Comparatively, respirators, which are also certified medical PPE and have a variety of ratings (of which N95 is the most commonly used in North America), are useful for user protection against small respiratory particles known as aerosols or droplet nuclei [9]. In both cases, the primary reason these PPE are used in healthcare is the protection of the wearer or inward protection. However, there is an additional role of both surgical masks and respirators to retain respiratory particles in order to avoid spread to others, also known as outward protection.

Prior to the COVID-19 pandemic, the usage of cloth masks in healthcare and the community is commonly observed in many Asian countries, including China and Vietnam [10,11]. During the severe acute respiratory syndrome (SARS) outbreak in 2002, there were reports of the usage of cotton cloth masks among HCWs in China [12]. In the current COVID-19 pandemic, Chinese recommendations on face mask use in community settings suggest that cloth masks could be used in a very low-risk population to prevent the spread of disease [13]. In the western world, the use of cloth masks is rarely witnessed in healthcare settings due to the availability of surgical masks and respirators. In times of a global pandemic with limited resources, cloth masks may be useful in protecting HCWs and retaining fluids and droplets in infected patients. However, there is a lack of comprehensive literature that summarizes the latest findings on the extended use and reusability of cloth masks [9] along with limited guidance on its use during the COVID-19 pandemic. This review aims to integrate current studies and guidelines to determine the efficacy of cloth masks as both inward and outward protective equipment and whether they can be used in healthcare settings and/or the community in light of the PPE shortage. Furthermore, evidence-based suggestions are made on the most effective use of cloth masks during the times of pandemic.

## Review

Methods

Search Strategy

The search strategy was conducted on March 31, 2020, using an open date search strategy. The search terms used were “masks”, “respiratory protective device”, “facemask” to capture articles studying face masks. The terms “cotton”, “cloth”, “homemade”, “home made”, “DIY”, “do it yourself”, “t-shirt”, “muslin”, “gauze”, “cheese cloth”, “towel”, “fabric”, “tight woven” and “tight weave” were used to find articles related to cloth masks. The search strategy was employed on Embase, Medline, and Google Scholar. The search strategy was updated on April 6, 2020.

The titles and abstracts obtained from search strategies were screened by three reviewers (C.X., S.K., M.J.). Discrepancies were resolved by discussion between the three reviewers. The same reviewers also completed the full-text review. The reference list of studies selected for the review was screened by one reviewer to gather additional articles.

 

Inclusion/Exclusion Criteria for Studies

Eligible criteria comprised randomized controlled trials (RCTs), observational studies, laboratory studies, case series, and case studies. All English language full-text articles conducted on humans were included regardless of the age of participants. The study intervention must have included a cloth mask made of cotton, gauze, silk, or muslin, or of materials commonly found in a household. Comparators for the intervention may include surgical/medical masks or N95 respirators, but a comparison group was not mandatory for inclusion. The study outcomes of interest about cloth masks were clinical efficacy, filtration efficacy of material used in the mask, compliance and comfort, side effects, reusability, and the fit of the mask.

Exclusion criteria included letters to the editor, conference abstracts or insufficient data, animal studies, and lack of primary data. There were no restrictions on the setting. Studies conducted prior to 1950 were excluded.

Guideline search strategy

We searched for guidelines relevant to cloth masks on March 31, 2020. The search terms used were “cloth”, “fabric”, “homemade”, “mask”, “face mask”, “Facemask”, “respirator”, “N95 respirator”, “PPE”, “personal protective equipment”, “protection”, “infection control”. Guidelines search was conducted in the published material by the WHO, CDC, Canadian Medical Association Infobase Clinical Practice Guidelines, Toward Optimized Practice Guidelines, Clinical Practice Guidelines and Protocols in British Columbia, U.S. Preventative Services Task Force, Canadian Task Force on Preventive Health Care, and NICE (National Institute for Health and Care Excellence). The search for guidelines was updated on April 7, 2020.

Results

Study Characteristics

**Figure 1:**
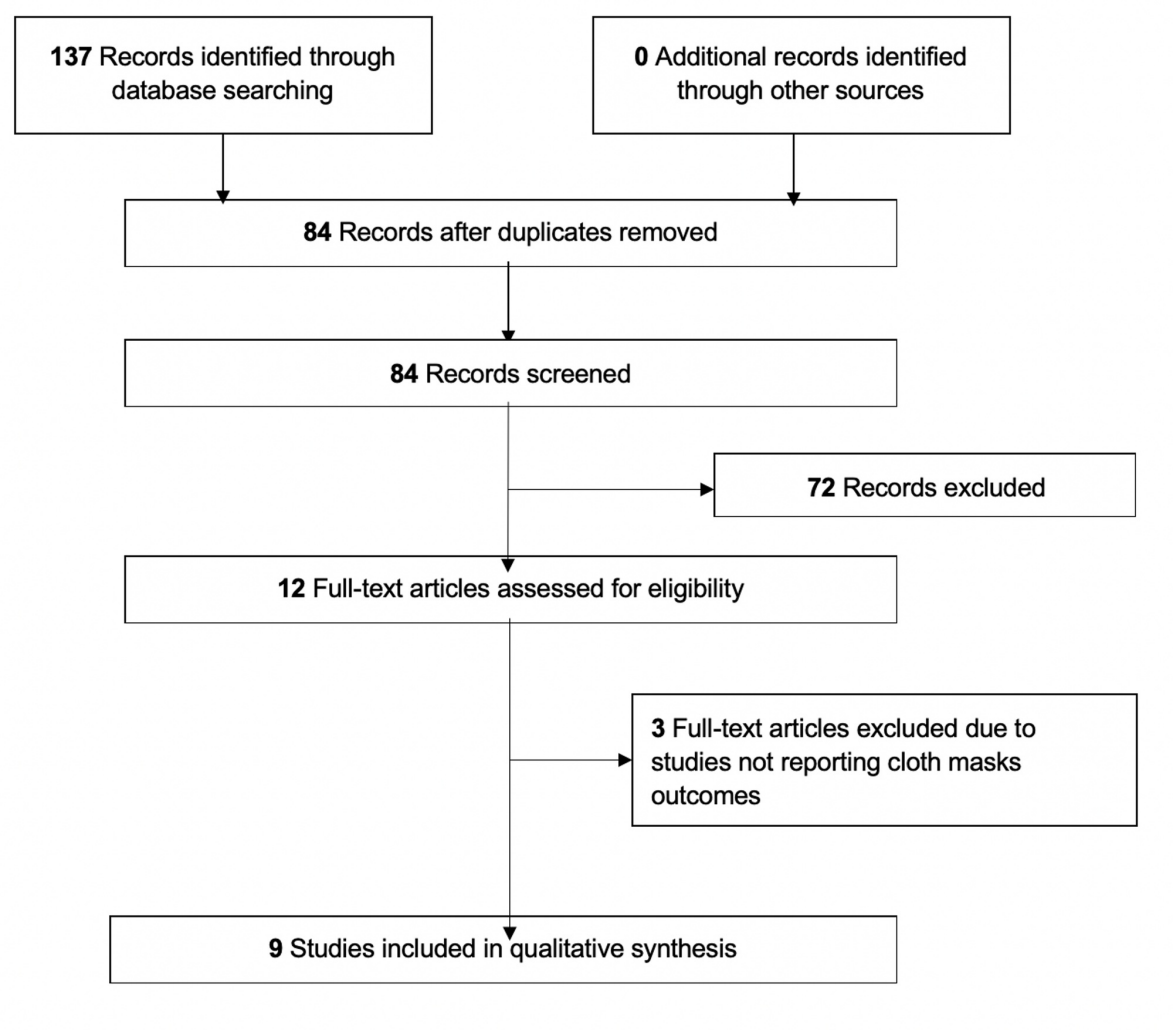
PRISMA flow diagram for the study selection process. PRISMA, Preferred Reporting Items for Systematic Reviews and Meta-Analyses

A search of databases yielded a total of 84 abstracts to screen. After title/abstract screening, 12 articles were selected for full-text review. Nine articles were included in this review after full-text screening (Figure 1) comprising one RCT [14], one observational study [10], five laboratory studies [15-19], and two studies that were a combination of observational and laboratory studies [20,21].

The study population comprised HCWs and healthy volunteers. Two studies, one observational and one RCT, were conducted on HCW participants [10,14]. Another three studies were conducted on healthy volunteers [18,20,21]. Of the nine studies, four used cotton cloth masks [10,14,17,18], one used polyester masks [19], and four [15,16,20,21] compared different types of materials commonly found in a home as possible materials for homemade masks.

The characteristics and results of each study are summarized in Table 1.

**Table 1:** Characteristics of included studies. HCW, healthcare worker; CRI, clinical respiratory illness; ILI, influenza-like illness; AIV, avian influenza virus

Author	Type of Study	Primary Outcomes	Methods	Participants	Type of Mask	Relevant Findings/Conclusions
Quesnel [18]	Laboratory study	Filtration efficiency; outward protection	Volunteers wore masks made of different materials, and contaminated particles escaping through the mask were measured in a sampling chamber	Volunteers	Cotton-muslin mask	Best performing masks contained more fabric, were softer, and pleated; reusable cotton masks as well as synthetic fiber masks also performed well when designed well
Furuhashi [17]	Laboratory Study	Filtration efficiency	A special apparatus was used to measure aerosol filtration of bacteria		Cotton mask	Bacterial filtration efficiency of cotton cloth masks was lower and more variable than surgical face masks
van der Sande et al. [21]	Laboratory study	Inward protection during short-term activities; inward protection during regular activities for longer duration; outward protection	Healthy volunteers used FFP2 (N95 equivalent), surgical masks, and homemade masks while performing a series of activities; particles on both internal and external sides of masks were measured by attached receptors and a portable particle counter; outward protection was tested using an artificial head attached to a respirator	Healthy volunteers	Homemade mask made of tea cloth	All types of masks provided protection against transmission for short- and long-term activities, with FFP2 being better than surgical masks and surgical masks being better than homemade masks; children are less protected than adults; no significant impact of activity/duration on protection; all types of masks provided reduced outward protection relative to inward protection; homemade masks conferred minimal amount of outward protection
Rengasamy et al. [16]	Laboratory study	Filtration efficiency of cloth masks and various materials against nanoparticles	Cloth masks and common fabrics were tested for polydisperse and monodisperse aerosols (20–1000 nm) at two different face velocities (5.5 and 16.5 cm s21) and compared with the N95 respirator filter		Sweatshirts, T-shirts, towels, scarves, and cloth masks	Higher penetration levels were observed for fabric materials than N95 respirator filter; common fabric materials may provide marginal protection against nanoparticles including virus-containing particles during expiration
Yang et al. [10]	Cross-sectional study	Rate of mask-wearing; rate of respiratory infections; other associated factors	HCWs in Beijing were surveyed using a standardized questionnaire	HCWs	Cotton-yarn mask	Wearing medical masks lowers the risk of respiratory infection in comparison to cotton-yarn masks; most commonly used masks are cotton-yarn masks; HCWs with good adherence to mask-wearing were at lower risk of respiratory infection
Davies et al. [20]	Laboratory study	Filtration efficiency and pressure drop of various household materials; fit; ability to prevent droplet/particle dissemination from the wearer	Healthy volunteers made their own face masks and were tested for fit; in a cough-box experiment, surgical masks, homemade masks, and no mask were compared for the number of microorganisms isolated from coughs of healthy volunteers	Healthy volunteers	Homemade masks made of cotton T-shirts	Pillowcases and cotton T-shirts were the most suitable household materials for face masks, considering both filtration efficiency and pressure drop; homemade masks reduced the total number of microorganisms expelled by coughing, but surgical masks were found to be more effective, especially for smaller particles; homemade face masks may be considered for HCWs who are at increased risk due to frequent contact with patients in resource-limited settings
MacIntyre et al. [14]	Randomized controlled trial	CRI; ILI; laboratory-confirmed viral infection; compliance	Cluster randomization of HCWs to cloth mask group, medical mask group, and control group (standard practice) and measured primary outcomes after 4 weeks of mask use and 1 week for appearance of symptoms	HCWs	Double-layered cotton mask	Cloth mask arm showed the highest rate of CRI, ILI, and laboratory-confirmed viral infections followed by the control arm and lowest in the medical mask arm; ILI was significantly higher in the cloth mask group compared to medical mask and control groups
Neupane et al. [15]	Laboratory study	Surface characterization; filtration efficiency; effect of washing, drying, and stretching on filtration efficiency	The surfaces of cloth masks were characterized using optical image analysis; filtration efficiencies were measured using the particle counting method		Double-layered cloth mask	Filtration efficiency of cloth masks is lower than surgical masks; washing, drying, or stretching reduces filtration efficiency of cloth masks by changing the pore size and shape; washing reduces the number of microfibers in the pores
Ma et al. [19]	Laboratory study	Filtration efficiency; removal of virus by hand-washing	Cloth mask, surgical mask, and N95 respirators were exposed to AIV in aerosols; the percentage of blockage of AIV was measured		Homemade mask made of four-layer kitchen paper and one layer cloth	All three types of masks could block AIV effectively, with N95 respirators being the most effective followed by surgical masks; mask-wearing and instant hand hygiene may prevent the spread of COVID-19

Clinical Efficacy

Three studies measured inward protection of cloth masks in human subjects [10,14,21]. Out of three studies, one RCT showed that the cloth mask group had the highest rate of influenza-like illness compared to the medical mask group and control group and cautioned that cloth masks should not be recommended for HCWs in high-risk settings [14]. However, the results from this study are difficult to interpret as the control group was “standard practice”, comprising individuals using both medical and cloth masks. One other study showed that homemade masks made of tea cloth provided protection during short- and long-term activities compared to no mask [21]. Ma et al. showed that while N95 respirators blocked 99.98% avian influenza virus, cloth homemade masks and surgical masks were comparable (95.15% and 97.14%, respectively). These homemade masks used in the experiment were made from polyester and kitchen towels [19]. Three articles showed that cloth masks resulted in higher rates of infection or particle exposure as compared to surgical masks [10,14,21].

Three studies specifically measured outward protection either with human subjects [18,20] or by simulating expiration with an artificial head [21]. In human subjects, both surgical and cloth masks were effective in controlling the number of microorganisms released into the environment when coughing, though surgical masks were more effective, especially with smaller particles [20]. In an older study, Quesnel showed that a cotton mask, which was not homemade, provided equivalent outward protection as two other surgical masks [18]. In an experimental setup with an artificial head, cloth masks provided marginal outward protection [21].

Filtration Efficacy

A few studies compared the filtration efficacy of various household materials [16,20]. One such study assessed pressure drop across different household materials to assess comfort of material when used in the masks along with filtration efficiency against microbial aerosols. Davies et al. used both *Bacillus atrophaeus* (0.95-1.25 um) and *Bacteriophage MS2* (23 nm) to generate microbial aerosols for the simulation of particle challenge. They found pillowcases and 100% cotton T-shirts to be most suitable to construct more efficacious cloth masks compared to tea towels, vacuum cleaner bags, silk, and so on [20]. Another laboratory study evaluated the penetration of monodispersed NaCl aerosol particles through cloth masks made of various materials (sweatshirts, T-shirts, towels, or scarves). The penetration of these masks was 35-68% at 20 nm in diameter and 73-82% at 100-400 nm [16]. Assuming that SARS-CoV2 particles are of a similar size as SARS-CoV particles from the 2002-2004 outbreak (80-140 nm), these nanoparticles are in the relevant size range [22]. Studies that compared filtration efficacy of cloth masks to surgical masks or N95 respirators found that particle penetration was consistently higher in cloth masks [14,15]. Another study showed no significant difference in the efficacy of surgical masks compared to well-constructed reusable four-ply cotton muslin masks when testing micro- and nanoparticles together [18].

Compliance

Higher compliance with cloth masks is seen in low- to middle-income countries and during pandemics due to the overall lack of PPE [10,15,20]. During the H1N1 pandemic, the majority of doctors and nurses used cloth masks (self-reported: 59.8%) over medical masks across eight hospitals in Beijing, China [10]. Another study reported that HCWs showed equal compliance when wearing cloth as compared to medical masks (57%), where compliance was defined as wearing the mask more than 70% of the time [14]. The main adverse events that decreased compliance were general discomfort and difficulty breathing, though adverse events were reported in both medical and cloth mask groups (40.4% and 42.6%, respectively) [14]. In Kathmandu, Nepal, 31% of the general population surveyed were found to wear cloth masks on the streets to protect themselves against pollution [15].

Fit Testing

The fit of a mask is an important variable in determining its efficacy. It is considered an area of weakness for cloth masks. Davies et al. used the Wilcoxon sign rank test to assess the fit of surgical and cotton cloth masks. The participants underwent a variety of head and body movements while wearing the masks, and fit testing was also performed at rest. They determined the fit of surgical masks to be significantly superior (P < 0.001) than cotton cloth masks in all activities and at rest [20].

Reusability

Some studies reported reusability and resulting contamination of cloth face masks; however, only one study quantified this. This study showed a negative linear trend between washing and drying cycles and filtration efficacy (*R*^2^ = 0.99). After the fourth wash and dry cycle, the efficacy of the mask had decreased by 20%. Microscopic imaging of these masks after wash and dry cycles showed an increase in pore size, change in pore shape, and decrease in the number of microfibers in each pore after these cycles [15].

Guidelines

There are no current guidelines or standardized protocols on the use or creation of cloth masks. The WHO presented interim guidelines in March 2020 in the context of the COVID-19 pandemic stating that they do not recommend the use of cloth masks in healthcare settings, in the community, or at home [23]. Another set of recommendations from WHO published on April 6, 2020, also stated that cloth masks are not appropriate for HCWs. If cloth masks are used locally, the WHO highly encourages local authorities to assess the masks [24]. The CDC suggests that HCWs use homemade masks if certified face masks are not available. However, they state that these masks are not considered PPE. The CDC also recommends that homemade masks should be used with a face shield covering the entire face [25]. Furthermore, on April 3, 2020, the CDC released recommendations asking the general population to wear cloth masks in areas where socially distancing is not possible [26]. They also released tutorials on how to create these masks [27].

Discussion

To our knowledge, this is the first review to descriptively synthesize and evaluate the best available evidence on the efficacy of cloth masks, providing relevant and useful information that can guide public health guidelines during the current COVID-19 pandemic. To date, there are little data to make definite recommendations as only one RCT [14] and a few observational studies [10,20,21] have been conducted on this topic. When assessing the overall clinical efficacy of cloth masks compared to surgical masks, two factors must be considered: inward and outward protection. The general consensus of the included studies is that cloth masks confer some degree of inward and outward protection, but are less effective than surgical masks and N95 respirators [10,14,20,21].

The clinical efficacy of a face mask is determined by the filtration efficacy of the material, fit of the mask, and compliance to wearing the mask [21]. Filtration efficacy of a material is the ability to function in both inward and outward protective gear. In general, household fabrics such as cotton T-shirts and towels [16,20] have some filtration efficacy and were shown to have some protection against virus-containing particles. However, the percentage of penetration in cloth masks was higher than surgical masks or N95 respirators. One study, however, suggested that a reusable cloth mask can have the same filtration efficacy as a surgical mask (98.8%) [18].

Surgical and cloth masks provide less outward protection partly due to the weaker seal around these masks. When pressurized droplets or aerosolized particles are released from the user (e.g., during a cough or sneeze), these particles have a higher likelihood of escaping from the sides than the front of the mask due to the mask’s fit. Cloth masks are inferior to surgical masks or N95 respirators when assessing the fit of the mask [20]. There is greater opportunity for air leakage around the sides of a cloth mask than the other two mask types, which decreases its ability to contain particles released by the user. However, Dato et al. showed a reasonable fit of their homemade mask in a letter to the editor of Emerging Infectious Disease. They presented a protocol for homemade 100% cotton masks that yielded a fit factor up to 67 (N95 respirators must have a fit factor of at least 100). Their homemade mask provided significant protection in an aerosol challenge. The recommended use of these masks was in situations where N95 respirators were unavailable [27].

Compliance of cloth masks does not differ from that of medical masks, indicating that homemade masks or masks of varying household fabrics are not any less comfortable. The main side effects were difficulty breathing and general discomfort, which were not unique to cloth masks [14]. In fact, in low- to middle-income countries, compliance may be higher due to a lack of availability of surgical masks. A study was conducted on focus groups of doctors and nurses in Vietnam to assess their compliance and opinions of face masks. The groups reported both cloth and medical masks to be comfortable to breathe through. Surgical masks were found to be associated with words such as “safe” and “effective”, whereas cloth masks were associated with “dirty” [28]. Given the variety of options available for different types of cloth masks, all that have shown comparable efficiency [15] while also allowing users to exert their preference and pick a material more comfortable to them.

Of the various sources searched, guidelines on the use and efficacy of cloth masks were limited to the WHO and CDC’s commentary on cloth masks not qualifying as PPE and the CDC’s suggestion of the general population using homemade masks [23,25,26]. The WHO and earlier CDC [23,24] guidelines focused on the usage of cloth masks as PPE to protect the user from the environment (inward protection) and did not address the use of cloth masks to contain droplets and secretions produced by infected individuals (outward protection). Cloth masks showed some evidence of outward protection [20] and filtration against microbial aerosols and nanoparticles [15-17,19,20], albeit in varying degrees, depending on the material. As a result, the potential for outward protection of cloth masks in healthcare settings should be better assessed and addressed in international guidelines.

There have been other guidelines posted on the websites of the WHO, CDC, and Canadian government, which suggest that cloth masks can aid in covering the mouth and nose when coughing [23,25,29]. Wearing a mask as prophylactic protection against a cough serves as better source control compared to finding mouth coverings spontaneously as needed. It should be noted that CDC recommended disposing of materials sneezed into [26]. Cloth masks can be cleaned to address this point in the guidelines. The Government of Canada also recommended the use of cloth masks by the public in situations where social distancing is not possible and stated that homemade cloth masks are not a replacement for surgical masks [30]. Moreover, British Columbia Centre for Disease Control (BCCDC) guidelines state that contaminated cloth can be cleaned with other pieces of clothing in a laundry machine. Hot water (60-90°C) with soap should be used to clean the laundry machine [26].

Many low- and middle-income use cloth masks in healthcare settings due to a lack of financial resources to support the wide use of surgical masks. Recommendations regarding cloth mask use in Vietnam, Pakistan, and China include wearing them during low-risk activity (e.g., slashes of fluid or blood, bacterial infection) in the situation of the influenza season and a pandemic [31]. Therefore, cloth masks that are regulated may provide some protection against viruses and bacteria.

Another benefit of using cloth masks in healthcare or community settings is that the production of these masks can be outsourced to freelancers or volunteers in the community if a stringent and tested protocol is developed. For example, in the COVID-19 pandemic, the lack of face masks and other PPE has been a global concern. Michael Garron Hospital in Toronto, Canada, asked volunteers to create cloth masks at home for use in healthcare due to lack of face masks. The project has provided volunteers with a protocol to follow when making the mask, but whether this protocol has been studied is unknown [32]. There is a tested protocol available through Davies et al.’s research study. This group designed and studied a protocol for cotton cloth masks; however, this protocol was not widely implemented as an effort to standardize or certify commercially available cloth masks [20]. Moreover, the CDC has also released a tutorial on creating homemade cloth masks; however, the web article does not state if this protocol or recommended materials to make the mask have been tested [33].

Strengths and limitations

There are several strengths to this review. This review provides a unique detailed analysis of the various characteristics that contribute to droplet retention and mask efficacy, including the filtration efficacy of the material, fit of the mask, and compliance of the user. The strength of this review lies in its systematic search of multiple databases and search strategies developed and conducted in conjunction with a research librarian. Moreover, international and national guidelines were collected to present the real-world implementation of existing research on cloth masks.

There are also several limitations to consider. Firstly, the scope of the recommendations presented in this review was limited by the lack of data available on cloth masks. Only one RCT has been conducted to date and few observational studies exist. Included studies did not present a quantitative analysis of the filtration efficacy and penetration of materials commonly used in cloth masks or report on the number of layers of cloth material required for maximized benefit and comfort. This highlights important research questions that future high-quality studies should explore to increase our understanding of the efficacy and use of cloth masks. Secondly, the heterogeneity of the included studies notably precluded a meta-analysis. Future studies should focus on defining comparable outcomes. Another limitation includes the fact that our search criteria limited our review to focus only on published studies. By not including grey literature, the review potentially misses out on other perspectives and information about the usage of cloth masks.

Future studies should investigate the effectiveness of masks in reducing travel velocities and distances of droplets and aerosols during expiration and coughing, which may reduce the transmission of COVID-19. Secondly, studies should also investigate the ability of cloth masks to reduce virus transmission by preventing the user from touching their face or droplets from landing on naso-oral surfaces. Lastly, to support the CDC recommendation of only using homemade masks in a healthcare setting if a face shield is worn [25], studies should investigate the efficacy of cloth masks used with 3D-printed face shields. Both are easily producible in situations of PPE shortage such as the COVID-19 pandemic, and if proven to provide adequate protection for HCW, they can be easily be produced in bulk by the general public. Results from these studies may be used to guide recommendations on the use of cloth masks for the general public when social distancing measures are in place.

To better understand the role that cloth masks play in pandemics and infectious control generally, further RCTs must be conducted. However, a study by MacIntyre et al. highlights the ethical challenge in designing a RCT for mask use, as HCWs in the control group cannot be asked to wear a mask when working in high-risk situations [14]. As this RCT did not address outward protection, future studies should look at whether cloth masks worn by infected patients can protect the transmission of infection among HCWs by retaining droplets and fluids. Future studies should make evident whether they are studying inward or outward protection as this discrepancy was unclear in some studies.

## Conclusions

Cloth masks are shown to have limited inward protection in healthcare settings where viral exposure is high but may be beneficial for outward protection in low-risk settings and use by the general public where no other alternatives to medical masks are available. During unprecedented times, such as the COVID-19 pandemic, when some organizations like the CDC are suggesting the general population to use cloth masks in public settings, further studies on cloth masks are imperative. The current data are not enough to guide clinical decision-making. Given that cloth masks are used when the supply of surgical masks is low, it is important to assess the true efficacy of cloth masks compared to not wearing any masks.
